# Dataset of the*Emiliania huxleyi* abundance and phytoplankton composition in the Barents Sea in summer 2014–2018

**DOI:** 10.1016/j.dib.2020.106251

**Published:** 2020-09-02

**Authors:** Vladimir Silkin, Larisa Pautova, Marina Kravchishina, Vladimir Artemiev, Anna Chultsova

**Affiliations:** Shirshov Institute of Oceanology, Russian Academy of Sciences, Moscow, Russia

**Keywords:** Coccolithophores bloom, *Emiliania huxleyi*, Nutrients, CTD-data, Barents Sea

## Abstract

This data article contains data on the *Emiliania huxleyi* abundance, phytoplankton composition, in the Barents Sea in summer 2014–2018, and physical and hydrochemical parameters in summer 2017. The data are based on the samples collected on stations, where *E. huxleyi* blooms were recorded. The physical data included the water temperature, salinity, oxygen concentrations at the surface, and various depths. Data of nutrients concentrations included followed parameters: silicates, phosphates, nitrates, nitrites, ammonium, and dissolved inorganic nitrogen. The nutrients ratios are also given. Data of phytoplankton composition consists of the abundance of diatoms, dinoflagellates, coccolithophores, and small flagellates.

The data presented in this article are associated with the research article entitled “Interannual variability of Emiliania huxlei blooms in the Barents Sea: In Situ data 2014–2018” [1]. The related research article examines the influence of abiotic factors such as temperature, salinity, nutrients concentrations, and biotic factors (phytoplankton composition) on E. huxleyi abundance.

**Specifications Table**

**Table:** 

Subject	Oceanology
Specific subject area	Biooceanology
Type of data	Table Image
How data were acquired	SEM (VEGA-3sem TESCAN, Czech Republic) and Light Microscope (Ergoval, Karl Zeiss, Jena); CTD (SBE-19 Plus; Sea Bird Equipment); TechNicon II autoanalyzer
Data format	Raw Analyzed
Parameters for data collection	All data were obtained in the Barents Sea during cruise 127 of the R/V *Professor Shtokman*, which took place from July 26 to August 5, 2014; cruises 62, 65, 68 and 71 of the R/V *Akademik Mstislav Keldysh*, which took place from July 22 to August 18, 2015, from June 29 to July 9, 2016, from July 23 to August 18 2017, and August 12–13 2018 respectively (Fig. 1 and Table S1, Supplementary material, original research article).
Description of data collection	Sampling was carried out from different depths, which was selected by CTD (SBE-19 Plus; Sea Bird Equipment) probing. The samples were collected using a rosette probe sampler with a 5–10-L Niskin bottles. The seawater was neutralized using 40% formalin buffered with borax, with a final concentration in the sample of 0.8–1.0%. Phytoplankton was concentrated by sedimentation.
Data source location	The Barents Sea; 40 stations between 21° 51.70–44° 20.94 E and 68° 01.1–75° 09.48 N
Data accessibility	Relevant data reported in this article related research article “Interannual variability of *Emiliania* huxleyi blooms in the Barents Sea: *In Situ* data 2014–2018” [1].

## Value of the Data

• Coccolithophores blooms in the Barents Sea usually study by remote sensing. However, this method cannot use for a precise estimate of the coccolithophores' abundance. Therefore satellite observations should be supported by *in situ* measurements that are rarely in the Barents Sea. This dataset provides precise information about the abundance of the coccolithophores and will be useful for the validation of satellite data.
• Coccolithophorids are responsible for the functioning of the carbonate pump in the ocean. These data provide complete information about abiotic and biotic factors, making it possible to determine the mechanisms of regulation of the carbonate pump. The primary beneficiaries of the data may be researchers working on the problem of the biological carbon pump in the ocean.
• Climate change in the Barents Sea is usually associated with increased intrusion of Atlantic waters. Their biological indicator is *Emiliania huxleyi*. This dataset provides accurate data on the northward movement of this species. Therefore, the data presented can be useful for researchers working on climate change in the Arctic region.

## 1 Data Description

**Fig. 1:**
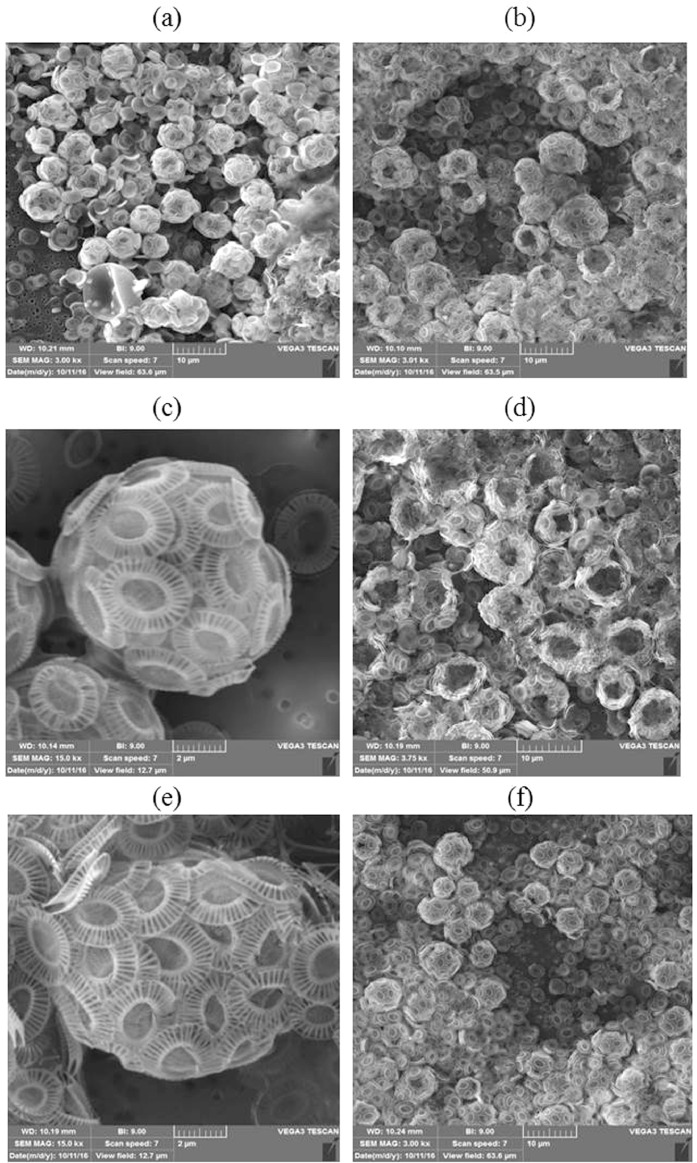
SEM photomicrographs of the *Emiliania huxleyi* in the southern part of the Barents Sea in July 2016: (a) station 6526, layer 0 m; (b) and (c) station 6534, layer 0 m; (d) station 6534, layer 10 m; (e) and (f) station 6534, layer 25 m. Station locations are shown in the Fig. 1 and Table S1, Supplementary material, original research article.

The data presented in this article hosts 2 tables and 1 figure (6 images). Table 1 hosts raw data about temperature, salinity, oxygen concentrations, nutrients concentrations (silicates, phosphates, nitrates, nitrites, ammonium, dissolved inorganic nitrogen), and their ratios and *Emiliania huxleyi* abundance in summer 2017 in the Barents Sea. This table shows only data at stations and at the depth where *Emiliania huxleyi* blooms were registered i.e. where the abundance exceeded 10^6^ cells per liter. Data of phytoplankton composition in the Barents Sea in 2014–2018 were presented in Table 2 where followed parameters were included: the abundance of diatoms, dinoflagellates, coccolithophores, and small flagellates. The Fig. 1 contains images (a)–(f) show SEM microphotographs of *E. huxleyi* received on the data of cruise 67 of the R/V *Akademik Mstislav Keldysh* in summer 2016. These images demonstrate the variability of cells and coccoliths size during *E. huxleyi* bloom.

**Table 1:** . Temperature, salinity, dissolved oxygen, nutrients concentrations and their ratios and *Emiliania huxleyi* abundance in the Barents Sea in August 2017.

Station	Depth	T	S	О_2_	О_2_	Si	P−PO_4_^−^	$N-{\text{NO}}_{2}^{-}$	$N-{\text{NO}}_{3}^{-}$	N−NH_4_^+^	DIN	DIN:P	Si:DIN	Si:P	*E. huxleyi*
	m	°C	*psu*	ml/L	%	μM	μM	μM	μM	μM	μM				10^6^ cells/L
5544	5	7.32	35.02	7.19	108	0.24	0.03	0.02	0.06	0.48	0.56	19.81	0.42	8.29	1.82
5548	5	7.56	34.98	7.41	111	0.28	0.08	0.01	0.13	0.64	0.78	10.34	0.36	3.73	1.15
5550	5	7.15	35.00	7.35	109	0.24	0.07	0.02	0.15	0.75	0.92	13.86	0.26	3.55	1.15
5548А	5	7.44	34.94	7.08	106	0.42	0.07	0.02	0.12	0.64	0.78	11.82	0.54	6.39	0.98
5548А	20	7.44	34.97	7.09	106	0.42	0.07	0.02	0.21	0.59	0.82	12.31	0.52	6.39	1.56
5574	5	8.26	34.93	6.79	103	0.85	0.13	0.06	0.71	0.91	1.68	12.66	0.50	6.39	1.44
5576	5	9.38	34.50	6.63	103	0.71	0.12	0.03	0.15	0.64	0.81	6.60	0.87	5.74	6.62
5577	5	9.31	34.64	6.71	90	0.42	0.04	0.02	0.18	1.02	1.22	32.05	0.35	11.19	4.10
5578	5	10.09	34.18	6.65	87	0.19	0.05	0.02	0.26	1.07	1.35	28.58	0.14	3.98	3.17
5579	5	10.72	33.91	6.59	102	0.05	0.04	0.01	0.19	0.64	0.84	22.19	0.06	1.24	3.26
5579	17	10.06	34.11	6.73	105	0.28	0.09	0.04	0.63	0.64	1.31	13.80	0.22	2.98	2.64
5580	10	9.79	34.45	6.67	105	0.28	0.09	0.02	0.10	0.53	0.65	7.60	0.44	3.31	5.28
5580	22	9.21	34.45	6.73	104	0.28	0.08	0.03	0.29	0.75	1.07	14.07	0.26	3.73	3.65
5581	5	8.64	34.66	6.95	106	0.47	0.05	0.02	0.18	0.80	1.00	21.13	0.47	9.94	1.62
5581	15	8.66	34.66	6.74	103	0.42	0.09	0.02	0.15	0.69	0.87	10.15	0.49	4.97	1.94
5581	22	7.32	34.65	7.01	104	0.90	0.13	0.03	0.23	1.02	1.28	9.62	0.70	6.75	2.42

**Table 2:** Phytoplankton abundance (cells/L) of different taxonomic and size groups in the Barents Sea in 2014–2018 at the stations where *Emiliania huxleyi* bloom was observed.

Station	Depth, m	Diatoms	Dinoflagellates	*E. huxleyi*	Small flagellates
2014					
33	0	0	235	1,391,200	689,000
33	4	0	400	1,548,000	828,000
34	0	51	15500	3,060,000	448,800
34	4	0	6900	1,800,000	288,000
35	0	0	23800	6,936,000	1,101,600
35	4	0	2100	8,347,200	689,000
36	0	0	3670	2,448,000	244,800
36	4	0	14500	3,196,000	300,800
2015					
5192	0	0	1860	2,889,600	598,600
5193	23	0	970	2,476,800	591,700
5195	0	0	1980	1,428,000	319,600
5195	10	0	5670	3,264,000	877,200
5195	21	0	5700	1,224,000	584,800
2016					
25	0	0	7330	4,071,000	50,900
26	0	0	740	5,391,500	610,900
30	0	0	300	7,505,555	40,200
31	0	9	1900	12,000,000	44,700
32	0	9	230	4,145,000	24,000
33	0	0	855	5,091,000	39,800
33	5	18	2055	8,000,000	50,900
33	19	9	170	7,854,500	47,800
34	0	0	760	9,309,100	38,200
34	10	0	1445	9,454,500	102,000
34	25	0	380	3,636,000	30,000
35	0	0	245	1,818,200	80,000
2017					
5544	5	29	62800	1,746,000	11,100
5548	5	0	6360	1,097,000	315,000
5550	5	0	54600	1,097,000	185,000
5548-A	20	6	117400	1,490,000	22,900
5574	5	96	1630	1,371,000	45,700
5576	5	217	2100	6,308,600	59,000
5577	5	0	1500	4,114,000	27,000
5578	5	21600	11700	3,017,000	12,800
5579	5	28800	1570	3,108,571	11,000
5579	17	11440	1680	2,514,000	12,000
5580	10	0	2040	5,029,000	22,900
5580	22	0	4360	3,474,000	6000
5581	5	69	1080	1,555,000	197,000
5581	15	69	1150	1,85,2000	93,600
5581	22	801	1030	2,309,000	119,000
2018					
5940	0	0	930	1,440,000	144,000
5940	2	9	1770	1,426,000	67,000
5940	5	9	1330	1,385,000	41,000
5940	10	0	1800	1,645,700	11,2000
5940	23	26	690	1,728,000	68,600
7104	0	44	2860	1,101,000	967,600
7106	0	77	740	1,097,000	75,000
7107	0	86	620	1,406,000	54,900
7108	0	0	420	2,112,000	29,5000

## 2 Experimental design, materials and methods

### 2.1 Sampling

All data were obtained in the Barents Sea during cruise 127 of the R/V *Professor Shtokman*, which took place from July 26 to August 5, 2014, cruises 62, 65, 68 and 71 of the R/V *Akademik Mstislav Keldysh*, which took place from July 22 to August 18, 2015, from June 29 to July 9, 2016, from July 23 to August 18 2017, and August 12–13 2018 respectively. Coordinates of 40 sampling stations were situated between 21° 51.70–44° 20.94 E and 68° 01.1–75°09.48 N (Table S1, Supplementary material, original research article). In 2014, water samples were taken from the water surface and at a depth of 4 m. In 2015, 2016, 2017 and 2018, sampling was carried out from different depths. Temperature and salinity were measured by using CTD device (SBE-19 Plus; Sea Bird Equipment). The water samples were collected using a rosette probe sampler with a 5–l0-L Niskin bottles. The seawater was fixed using neutralized 40% formalin buffered with borax, with a final concentration in the sample of 0.8–1.0%. Phytoplankton was concentrated by sedimentation.

### 2.2 Species identification and phytoplankton cells counting

Species identification was based on morphology, according to [2,3] and the World Register of Marine Species (http://www.marinespecies.org). The identification and counting of cells were conducted using a light microscope Ergoval (Karl Zeiss, Jena), with 16 × 10 and 16 × 40 magnifications. Cells with linear dimensions below 20 µm were counted using a Naujotte chamber (0.05 ml); the larger cells were counted using a Naumann chamber (1 ml). Unidentified species of the size group 4–10 µm were assigned to the group of small flagellates. Cells with linear dimensions smaller than 2 µm were not taken into account for the estimation of total phytoplankton biomass. Cell biovolume and biomass was estimated, according to [4,5]. Converting wet phytoplankton biomass to carbon units were carried out by using allometric equations [6].

### 2.3 SEM microphotographs of Emiliania huxleyi

Microphotographs of *E. huxleyi* was received by using VEGA-3sem TESCAN (Czech Republic) scanning electron microscopy (SEM) (Fig. 1).

Water samples were filtered through membrane filters (0.45 μm pore size) using a vacuum pump (under a vacuum ≤ 400 mbar) immediately after sampling in July 2016. Then samples were dried at *T* = 50 °C and transported to the lab and examined using SEM. For observation in secondary electron (SE) mode, the samples were coated with a 3- to 5-nm-thick gold layer.

### 2.4 Chemical analyses

Analyses of nutrients (phosphate, silicate, nitrate, nitrite, and ammonia) were carried out in 2016–2017 with a segmented continuous-flow TechNicon II autoanalyzer or with colorimetric methods [7,8] (Table 1). Dissolved inorganic nitrogen (DIN) was as the sum of nitrate, nitrite, and ammonia. Dissolved oxygen was analyzed by the Winkler method.

### 2.5 Determination of phytoplankton composition

Using a light microscope, cells abundance of various species was calculated. All species were divided into three taxonomic groups (diatoms, dinoflagellates, and coccolithophores) and one size group (small flagellates) (Table 2). This group included non-identified cells with linear sizes from 4 to 10 microns.

## Declaration of Competing Interest

The authors declare that they have no known competing financial interests or personal relationships which have, or could be perceived to have, influenced the work reported in this article.

## References

[1] Silkin V., Pautova L., Giordano M., Kravchishina M., Artemiev V. (2020). Interannual variability of *Emiliania huxlei* blooms in the Barents Sea: In Situ data 2014–2018. Mar. Pollut. Bull..

[2] Tomas C.R. (2007). Identifying Marine Phytoplankton.

[3] Throndsen J., Hasle G.R., Tangen K. (2007). Phytoplankton of Norwegian Coastal Waters.

[4] Hillebrand H., Durselen C., Kirschtel D., Pollingher U., Zohary T. (1999). Biovolume calculation for pelagic and benthic microalgae. J. Phycol..

[5] Moncheva S., Parr B. (2010). Manual for Phytoplankton Sampling and Analysis in the Black Sea. http://www.blacksea-commission.org/Downloads/Phytoplankton_%20Mannual-Final-1.pdf.

[6] Menden-Deuer S., E.J. Lessard (2000). Carbon to volume relationships for dinoflagellates, diatoms, and other protist plankton. Limnol. Oceanogr..

[7] O.K. Bordovskiy, A.M. Chernyakova (Eds.), Modern Methods of the Ocean Hydrochemical Investigations, P.P. Shirshov Institute of Oceanology: Moscow, 1992. (In Russian).

[8] Grasshoff K., Kremling K., Ehrhardt M. (2007). Methods of Seawater Analysis: Third, Completely Revised and Extended Edition. Methods of Seawater Analysis: Third, Completely Revised and Extended Edition.

